# Pigment Epithelium-Derived Factor Released by Müller Glial Cells Exerts Neuroprotective Effects on Retinal Ganglion Cells

**DOI:** 10.1007/s11064-012-0747-8

**Published:** 2012-03-13

**Authors:** Jan Darius Unterlauft, Wolfram Eichler, Konstantin Kuhne, Xiu Mei Yang, Yousef Yafai, Peter Wiedemann, Andreas Reichenbach, Thomas Claudepierre

**Affiliations:** 1 Department of Ophthalmology and Eye Hospital, University of Leipzig, Liebigstrasse 10-14, 04103 Leipzig, Germany; 2 Paul Flechsig Institute of Brain Research, University of Leipzig, Jahnallee 59, 04109 Leipzig, Germany

**Keywords:** Neuroprotection, Neuroregeneration, Neuron-glia interaction, Reactive glia

## Abstract

Survival of retinal ganglion cells (RGC) is compromised in several vision-threatening disorders such as ischemic and hypertensive retinopathies and glaucoma. Pigment epithelium-derived factor (PEDF) is a naturally occurring pleiotropic secreted factor in the retina. PEDF produced by retinal glial (Müller) cells is suspected to be an essential component of neuron-glial interactions especially for RGC, as it can protect this neuronal type from ischemia-induced cell death. Here we show that PEDF treatment can directly affect RGC survival in vitro. Using Müller cell-RGC-co-cultures we observed that activity of Müller-cell derived soluble mediators can attenuate hypoxia-induced damage and RGC loss. Finally, neutralizing the activity of PEDF in glia-conditioned media partially abolished the neuroprotective effect of glia, leading to an increased neuronal death in hypoxic condition. Altogether our results suggest that PEDF is crucially involved in the neuroprotective process of reactive Müller cells towards RGC.

## Introduction

Pigment epithelium-derived factor (PEDF) is a 50 kDa glycoprotein that belongs to the non-inhibitory serpin family [1] and is a key anti-angiogenic molecule [2, 3]. In retina it has been shown that PEDF is not only produced by the pigmented epithelium [4] but also by Müller glial cells [5, 6] and that it can counterbalance elevated levels of pro-angiogenic vascular endothelial growth factor (VEGF) [7, 8] In the eye, PEDF is therefore critically involved in the pro-/anti angiogenic balance provided by reactive glia in response to a hypoxic insult [9, 10]. In addition, PEDF has been recognized as an important endogenous anti-oxidative and anti-inflammatory factor [11–13]. Vitreous levels of PEDF were significantly decreased in proliferative diabetic retinopathy, suggesting that reduction of PEDF in the retina may contribute to the pathogenesis of this disease by worsening the retinal inflammation [12, 14]. PEDF not only suppresses pathological neovascularization and inflammation, but in addition to its anti-angiogenic and anti-inflammatory properties PEDF exerts neuroprotective and neurotrophic effects [15, 16] that are mediated by activation of NF-κB and ERK−1/−2 pathways [17]. In retina PEDF protects neurons from light-induced damage, oxidative stress [18], as well as from apoptosis in mouse models of inherited retinal degeneration [19, 20]. PEDF has also been shown to protect rat retinal ganglion cells (RGC) from glutamate- to trophic factor withdrawal-mediated cytotoxicity [17]. In addition, PEDF protects RGCs from ischemia-induced neuronal cell death, reducing RGC loss [21]. Moreover, Zhou et al. [22] showed that PEDF reduces RGC loss and vision decline in DBA/2 J mice [22], a mouse model for inherited glaucoma [23, 24], the second leading causes of blindness in human [25]. PEDF-induced neuroprotection of RGC may therefore represent a promising strategy in management of various pathological states targeting specifically this neuronal type [26].

However, even if there are evidence for PEDF receptors at RGC surface [27, 28] no data exists concerning the direct effect of PEDF on this neuronal type. Most of the literature focuses either on global neuroprotection in vivo [22, 29] or consists in PEDF treatment of retinal mixed cultures in which RGC neurons represent only a small fraction of the total cells [17, 18]. It is therefore unclear whether RGC can directly respond to the PEDF stimulus or whether this effect is mediated via another cell type. In the latter case, survival of RGC would be an indirect consequence of the PEDF release. This prompted us to study these unknown aspects by treating pure RGC cultures with PEDF and analyzing its effects on neuronal survival in normal and hypoxic conditions.

The specific role of glial PEDF in the retina is also unclear as most of the earlier studies were based on intravitreal injections of PEDF protein or adenovirus-mediated delivery of the PEDF gene [21, 30, 31] barely affecting RGC which are separated from the retinal pigment epithelium (RPE) by the whole retinal thickness. Indeed the 50 kDa PEDF molecule secreted by RPE is theoretically able to cross the outer limiting membrane and all retinal layers, according to the permability properties of the retinal tissue [32], but injection of fluorescent-serpin clearly showed its accumulation in the photoreceptor layer whereas no signal was found in the RGC layer [33]. Therefore, to achieve PEDF-mediated neuroprotection of this particular neuronal type, another source is needed which could consist in Müller glial cells via their endfeet closely surrounding each RGC. Moreover, it has largely been documented that Müller glial cells are able to rapidly increase their level of PEDF release in response to ocular injury or hypoxia [8, 10, 34]. Therefore, PEDF might act more effectively on RGC when produced by their glial neighbour cells, and intraretinal PEDF levels governed by Müller cells may represent the very first line of defence against RGC aggressions. With this hypothesis in mind we have assessed the levels of PEDF expression in Müller cells as compared to other retinal cells. Using the model of pure RGC culture that we have developed recently from mice [35, 36] we further examined whether release of glia-derived PEDF is sufficient to achieve neuroprotective effects.

## Materials and Methods

### Materials

Supplements of culture media were from Sigma-Aldrich (Taufkirchen, Germany) unless otherwise indicated. Calcein-AM was from Molecular Probes, Inc. (Eugene, OR). PEDF originating from affinity-purified supernatants of transfected baby hamster kidney cells was provided by Millipore (Schwalbach, Germany).

### Animals

All animals were treated according to the association for research in vision and ophthalmology (ARVO) statement on the use of animals in ophthalmic and vision research, and all animal experiments were reviewed and approved by municipal and University Hospital animal care committees in Leipzig. TC is holding authorization #T97/11 for animal experimentation, delivered by the state of Saxony. P7 BALB/c mice and Long-Evan rats were obtained from the central animal facility of the medicine faculty in leipzig (MEZ). For RGC preparation newborn mice were rapidly decapitated, and for preparation of RPE and glial cells rats were euthanized using overdoses of carbon dioxide. Mouse and rats eyes were immediately collected and retina dissected thereafter.

### Immunoisolation and Culture of RGC from Postnatal Mouse Retinae

Seven days old mice were killed according to institutional guidelines. To isolate RGC, retinae were dissected and incubated for 45 min at 37°C in Dulbecco’s phosphate buffered saline (D-PBS; (Gibco/Invitrogen, Karlsruhe, Germany) containing 160 U/ml papain, 200 U/ml DNAse. The tissues were then sequentially triturated in D-PBS containing 0.15% trypsin inhibitor (Roche Diagnostics, Meylan, France), 650 U/ml DNAse and 1:75 rabbit anti-rat macrophage antibody. Cells were spun down (800′*g* for 13 min), resuspended in 1% trypsin inhibitor in D-PBS, spun down again and then resuspended in D-PBS containing 0.02% bovine serum-albumin (fraction V; Sigma). Cell suspension was filtered through a nylon-mesh (Nitex 20 μm, Tetko/Sefar Filtration, Rüschlikon, Switzerland) and sequentially added to immunopanning plates. Briefly, immunopanning was performed using two subtraction plates (150-mm diameter petri dishes; Falcon; BD Bioscience, Heidelberg, Germany) coated with goat anti-rabbit-IgG (Jackson Immunoresearch Laboratories/Beckman Coulter, Marseille, France; 10 μg/ml) to remove microglial cells after incubation of the supernatant with anti-macrophage antibody (WAK Chemie, Steinbach Germany). Positive cell isolation was then achieved by incubating the filtrated supernatant for 45 min on a 100-mm diameter petri dish sequentially pre-coated with goat anti-mouse-IgG (Jackson Immunoresearch Laboratories) and anti-Thy1.2 (mouse IgM, clone F7D5; Serotec, Düsseldorf, Germany). Non-adherent cells were thoroughly washed off, and the bound cells were released by trypsination (12,000 U/ml in Hank’s buffered salt solution [HBSS] for 10 min in 5% CO_2_ at 37°C) and resuspended in culture medium. The average yield, in thousands, of RGC per animal was 27.8 (±3.3), *n* = 11. RGC were plated at 600 cells mm^−2^ on glass coverslips (10 mm in diameter) centered on 12-well tissue culture plates (Greiner, Frickenhausen, Germany), which were pre-coated with 5 μg/ml poly-d-lysine (MW–40 kDa). Neurons were cultured for a week at 37°C, 5% CO_2_, 95% humidity in Neurobasal medium (Gibco/Invitrogen) supplemented with penicillin (100 U/ml)/streptomycin (100 μg/ml) and pyruvate (1 mM), glutamine (2 mM; Gibco/Invitrogen), *N*-acetyl-l-cysteine (60 μg/ml), putrescine (16 μg/ml), selenite (40 ng/ml), bovine serum albumin (100 μg/ml; fraction V, crystalline grade), triiodothyronine (40 ng/ml), holotransferrin (100 μg/ml), dibutyryl cyclic AMP (250 μM), insulin (5 μg/ml), progesterone (62 ng/ml), B27 (1:50, Gibco/Invitrogen), d-mannose (50 μM), brain-derived neurotrophic factor (BDNF; 25 ng/ml; PeproTech, London, UK), ciliary neurotrophic factor (CNTF; 10 ng/ml; PeproTech) and forskolin (10 μM). Cell viability was checked by examining the cell cultures under a phase contrast microscope (AxioVert25, Carl Zeiss, Jena, Germany) and determining cell adherence to the cover slip and the fraction of cells developing neurites. We waited a week in order to let RGC develop a massive neuritic arborisation. Since we were interested in the effect of PEDF on neuronal survival rather than on neurite growth, only preparations exhibiting an important development of neurites were selected for further tests.

### Isolation and Culture of Primary rat RPE and Retinal Glial Cells

Primary RPE cells were prepared from 6- to 9-days old rats as previously described [37]. The cells were isolated after mechanical removal and disruption of the RPE with subsequent enzymatic digestion using 0.05% trypsin/0.02% EDTA. RPE cells were cultured in a medium consisting of Ham’s-F10 supplemented with 10% fetal calf serum, 100 U/ml penicillin, and 100 μg/ml streptomycin. Primary retinal Müller cells were prepared from 6- to 9-days old rats as described previously [38]. Briefly, after animal death, eyes were removed and isolated retinae were dispersed for 30 min at 37°C in Ca^2+^- and Mg^2+^-free phosphate buffer supplemented with 1 mg/ml bacterial protease nagarse (subtilisin A, MP Biomedicals; Eschwege, Germany). After washing in phosphate buffer containing DNase I (200 U/ml), the dissociated cells were plated on glass coverslips and cultured in minimal essential medium supplemented with 10% FCS. Most of the cultured cells (~96%) expressed immunoreactivity for vimentin and were positively stained for glial fibrillary acidic protein.

### Homotyopic Culture, Co-culture and Hypoxia

#### Homotypic RGC Culture

After 1 week in vitro, the fully supplemented medium was changed to a minimal medium without growth factors for 24 h. Cells were then maintained under either normoxic (95% air, 5% CO_2_) or hypoxic conditions (0.2% O_2_, 5% CO_2_, 94.8% N_2_) for an additional 24 h in the minimal neurobasal medium, supplemented by 0, 1, 5 or 25 ng/ml PEDF or 10 ng/ml CNTF. The CNTF treatment was used as a control for optimal survival as its role in RGC neuroprotection has been largely demonstrated [35].

#### Co-culture

Primary rat Müller cells were cultured up to 90% of confluence. Prior to setup of the co-cultures, coverslips were briefly washed in HBSS (Invitrogen). Two cover slips with Müller cells (8.6 × 10^4^) and one with RGC (~5 × 10^4^) were co-cultured in a single 9.6 cm²-well of a 6-well plate (Greiner) for 24 h in a minimal neurobasal medium containing all previously cited components with the exception of BDNF and CNTF. This approach resulted in physical separation of the two cell populations but allowed access to media in each of the wells. Control experiments included homotypic RGC cultures. As we were interested in the neuroprotective role of glia-derived PEDF, co-cultures and homotypic RGC cultures were maintained in minimal neurobasal medium under either normoxic (95% air, 5% CO_2_) or hypoxic conditions (0.2% O_2_, 5% CO_2_, 94.8% N_2_) for an additional 24 h. Culture medium was supplemented by PEDF at different concentrations, CNTF (10 ng/ml) or preservative-free bioactivity-neutralizing rabbit anti-PEDF (PEDF1; 3 μg/ml; BioProducts Maryland, Middletown, USA) as indicated in the result section. Normal rabbit IgG (Dianova, Hamburg, Germany) was used as a control reagent.

### Survival Assays

RGC survival was assessed using a live-dead assay (Invitrogen) based on intracellular hydrolysis of calcein acetoxymethyl ester (calcein-AM) in living cells. Total number of cells was assessed by counter-staining with 4’-6-diamidino-2-phenylindole (DAPI). After incubation in normoxic or hypoxic conditions for 24 h, coverslips with RGC were incubated with 2 μM calcein acetoxymethyl ester for 30 min. The cells were washed using D-PBS, fixed in 4% paraformaldehyde solution for 15 min, washed 3 times in PBS before incubation in DAPI for 10 min followed by 3 final washes. Coverslips were mounted on microscope slides using Fluoromount G (Science Services, München, Germany). The slides were subsequently analyzed using a fluorescence microscope (AxioVision, Zeiss, Jena, Germany). Ten visual fields per coverslip were randomly chosen and the total amount of RGC and number of viable RGC per each field was assessed to give a survival percentage. We counted as living, cells labelled with both DAPI and calcein-AM exhibiting neurite arborisation and as dead, cells labelled only by DAPI and also cells exhibiting a pyknotic nucleus labelled with both DAPI and calcein, without neurite extension.

### Real-Time RT-PCR

Both RPE and Müller glial cells were passed at least once to avoid any neuronal contamination and cultured to confluence before RNA isolation. Total RNA from rat RPE and Müller cells was then prepared, treated with DNase I (Invitrogen), and subjected to reverse transcription using standard procedures. Aliquots of cDNA (2 μl) were amplified using a SYBR green assay (Bio-Rad, Munich, Germany). The PCR products were routinely analyzed by agarose gel electrophoresis and sequenced by intramural facility. Real time PCR included 0.2 μM of primers specific for DNA segments of rat PEDF (5′- CATGACATAGACCGAGAACTG -3′ and 5′- AAGGACTGTAGCTTCATGTCC -3′), or beta-actin (5′- GAAACTACATTCAATTCCATC -3′ and 5′- GGAGCAATGATCTTGATCTT -3). The PCR mix was denatured at 95°C for 6 min, followed by 45 cycles of melting at 95°C for 10 s, annealing at 58.5°C for 25 s, and elongation at 72°C for 25 s. All PCR data were checked for homogeneity by dissociation curve analysis. Fluorescence changes were monitored after each cycle, *C*t (threshold cycle) values for amplification of PEDF and β-actin mRNA were defined, and the level of PEDF mRNA in each sample was standardized to the endogenous β-actin level. Comparable efficiencies for PEDF and β-actin mRNA amplification were determined by analyzing serial cDNA dilutions. Relative PEDF mRNA were expressed as 2^−Δ*C*t^ with ΔCt = Ct_PEDF_–Ct_β-actin_.

### Immunohistochemistry

Adult mouse eye were prepared for cryosection by immersion in 4% formaldehyde solution in PBS for 1 h, followed by increased sucrose gradients (10, 20 and 30% in PBS). Eyeballs were embedded in TissueTek cryomedium (Miles Eckhart, USA). Cryosections (10 μm) were collected on Superfrost Plus (thermo fischer scientific) glass slides. Aspecific epitopes were blocked and cells were permeabilized using a solution of 30% casblock (Invitrogen) and 0.2% Triton ×100 in PBS for 30 min at RT. Primary mouse anti- cellular retinaldehyde binding protein (cralbp; NB100-74392 from Novus biological, Littelton, USA) and goat anti-PEDF (AF1149; R&D Systems Wiesbaden Germany) antibodies were used overnight at 4°C at 1/1,000 and 1/500 respectively in PBS, casblock 3, 0.02% triton ×100. Corresponding secondary antibodies coupled to Alexa 488 for anti-mouse and Alexa 555 for anti-goat were selected from Invitrogen and used at 1/500 for 1 h at RT. Finally, a DAPI (Invitrogen) nuclear staining was achieved in the last washing steps. Slides were mounted with Fluoromount-G (EMS, Hatfield, USA), observed using a Zeiss Axioplan 2 fluorescent microscope and picture taken with a Axiocam MRc5 digital camera coupled to AxioVison 4.6 software (Carl Zeiss). Control assays omitting primary antibodies confirmed the specificity of the staining (data not shown).

### Data Analysis

Each experiment was at least repeated 3 times per conditions. Bar diagrams display the means of RGC survival rate (±SEM). Comparisons between the means across all experimental conditions were made by analysis of variance (ANOVA). *P* < 0.05 was considered statistically significant.

## Results

### PEDF mRNA and Protein Expression in Glial Müller Cells and Effect of PEDF on RGC Survival

**Fig. 1:**
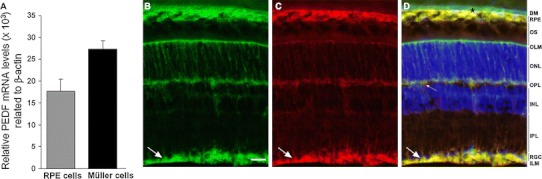
PEDF expression in rodent retina **a** relative expression of PEDF mRNA in rat Müller glia cells related to β-actin compare to rat RPE cells in vitro (in thousands). There was no statistical differences (*n* = 4) and therefore expression levels are comparable in both cell types in vitro suggesting that Müller glia cell is a source of PEDF within retina. **b**–**d** Immunohistological staining of mouse adult retina with antibodies against cralbp (**b**), and PEDF (**c**) and the resulting merged image **d** together with nucleus marker DAPI. Cralbp staining reveales the typical Müller glial cells morphology running radially through the entire retina thickness from the outer limiting membrane (OLM) to the inner limiting membrane (ILM) where the glia cells extend large end feet (*long arrow* in **b**–**d**) embedding the retinal ganglion cell (RGC) bodies. Retinal pigmented epithelium (RPE) cells are heavily stained with both cralbp and PEDF antibody, suggesting a high synthesis of this growth factor. Note the clear exclusion zone of PEDF staining above the RPE layer delimiting the Bruch’s membrane (BM, *black asterisk* in **d**). Within the neural retina, PEDF staining co-localizes mainly with cralbp in Müller glial cells. The signal is particularly strong at their end feet (*long arrow* in **b**, **c**, **d**) in close vicinity to RGC. In addition, in the outer plexiform layer corresponding to the photoreceptor synapses area, PEDF is seen in structures devoid of cralbp staining probably accounting for perivascular structure and/or extracellular deposit (*small arrow* in **c**, **d**). ONL: outer nuclear layer; INL: inner nuclear layer; IPL: inner plexiform layer; *scale bar* in **b**: 20 μm

**Fig. 2:**
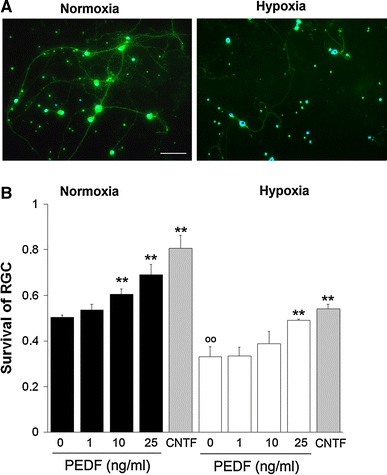
RGC survival under normoxia and hypoxia Survival of RGC under normoxia and hypoxia (0.2% O_2_). RGC were cultured for 24 h in a growth factor-deprived medium and survival was determined as indicated in the “Materials and Methods” section. **a** Representative sections of calcein-AM stained (*green*) RGC cultures maintained for 24 h under normoxia (*left panel*) or hypoxia (*right panel*) are shown. Cell nuclei were counterstained with DAPI (blue, *scale bar*, 50 μm). **b** PEDF enhances survival of RGC under normoxia and hypoxia. RGC survival (ratio of living cell vs. total cells) was measured after treatment with 0, 1, 5 and 25 ng/ml PEDF or 10 ng/ml CNTF (*n* = 3; treated versus normoxic or hypoxic PEDF-free cultures, *P < 0.05, **P < 0.01; control hypoxic versus normoxic PEDF-free control culture, ** ^°°^ *P* < 0.01). RGC cultures treated with CNTF (*bars in* *grey*) were taken as a reference for optimal survival

To evaluate the significance of Müller-cell related PEDF production in the retina we first compared their PEDF mRNA level to that of those cells thought to be main producers of PEDF in the eye, viz*.* RPE cells. Semi-quantitative real-time PCR analysis revealed that in vitro, level of PEDF mRNA in rat Müller and RPE cells were comparable (Fig. 1a). Relative PEDF mRNA levels (in thousands) related to β-actin were found to be 27.3 ± 2.0 in rat Müller cells and 17.6 ± 2.7 in rat RPE cells (*n* = 4). As we were testing in this study the effect of PEDF on mouse RGC we therefore analyzed the localisation of PEDF in mouse retina. We used the cralbp antibody to specifically label Müller glial cell within the retina. In addition to typical radial morphology of Müller cell in the retinal compartment, cralpb also label RPE and choroid (Fig. 1b). PEDF antibody revealed a highly similar localization. PEDF was found in cell structures running radially through the retinal layers from the outer to the inner limiting membranes (Fig. 1c). Merged image confirmed that, within the retina, PEDF was present in Müller glial cell (Fig. 1d). Signal was found especially strong at their large end feet (long arrow in Fig. 1c, d) in close contact with the nearby RGC, visualized using DAPI nuclear staining (Fig. 1d). PEDF staining was also found in the RPE but no signal was detected in the choroid as the limit of the double staining marked the Bruch’s membrane (asterisk in Fig. 1d). Based on those observations we then aimed to determine whether PEDF was able to exert a direct neuroprotective activity on pure RGC culture. We observed that hypoxia (0.2% O_2_) causes a compromised survival of RGC when stained by calcein-AM, which diffuses through cell membranes and is cleaved in the living cell by non-specific esterases to generate a fluorescent product, calcein. (Fig. 2a) When counting the number of calcein positive RGC versus the total number of cells in the field we found a ratio significantly reduced (^°°^ *P* < 0.01) in hypoxic condition versus normoxia (Fig. 2b). Under normoxia, RGC survival in absence of growth factor was 50.3 ± 0.1% and dropped to 33.0 ± 4.3%. However, addition of PEDF (range 0.02–0.5 nM [1–25 ng/ml]) to RGC cultures resulted in a dose-dependent increase of RGC survival, under normoxia (53.6 ± 2.5%; 60.4 ± 2.3%; and 69.1 ± 4.5% with 1, 5 and 25 ng/ml of PEDF respectively) that reached significance for the two higher doses of PEDF (**P < 0.01). This neuroprotective effect was also found under hypoxia (0.2% O_2_) as RGC survival increased along with PEDF concentration (33.3 ± 3.9%; 38.8 ± 5.4%; 49.0 ± 6.0%); however, survival rate was found significantly improved only with 25 ng/ml PEDF (**P < 0.01). CNTF was previously reported as a strong survival factor for RGC [35] and was used here to compare the efficiency of PEDF treatments. We found a survival of 80.7 ± 5.7% under normoxia and only 54.1 ± 1.9% under hypoxia that was significantly higher than control culture in normoxia and hypoxia (**P < 0.01). With the 25 ng/ml treatment we significantly increased the RGC survival in both normoxia and hypoxia. The survival rate did not reach a plateau in normoxia, however under hypoxia, 25 ng/ml PEDF had a nearly similar neuroprotective effect as CNTF treatment.

### Complex Secretions from Müller Cells Enhance the Viability of Retinal Ganglion Cells

**Fig. 3:**
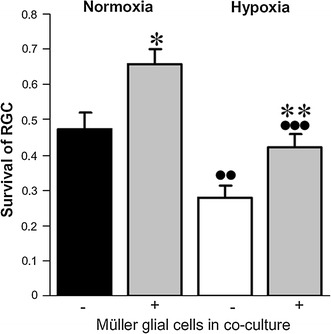
RGC and Müller glial cell co-cultures. Survival of RGC under normoxia or hypoxia (0.2% O_2_) and in the absence (−) or presence (+; *bars in* *grey*) of co-cultured primary Müller glial cells. Significant differences to cultures without Müller cells (*P < 0.05, **P < 0.01) and to corresponding normoxic conditions (^••^ *P* < 0.01, ^•••^ *P* < 0.001) are indicated (*n* = 6)

Given the deposition of RPE-synthesized PEDF in the interphotoreceptor matrix [39], we hypothesized that the majority of PEDF produced by RPE cells is barely accessible across the retina and thus would be an inappropriate means to protect RGC from survival-compromising insults. We therefore investigated the possibility that Müller-cell derived PEDF acts as a neuroprotectant for RGC. In an approach in vitro, we performed co-cultures of Müller cells and RGC under normoxia and hypoxia (0.2% O_2_), respectively, and determined RGC survival. Inspection of RGC after 24 h (*n* = 6) revealed 65.9 ± 4.4% and 42.2 ± 3.5% viable cells under normoxia and hypoxia, respectively, when the cells were co-cultured with primary rat glial (Müller) cells. In contrast, homotypic RGC cultures demonstrated a cellular survival amounting to 47.3 ± 4.8% and 27.9 ± 3.5% of the total cell number under normoxia and hypoxia, respectively. Thus the survival rate of RGC was significantly (*P* < 0.05) higher when cultured together with Müller cells than alone, each under normoxia and hypoxia. Also, in hypoxia we have consistently detected a significantly (*P* < 0.05) reduced number of viable RGC even in co-culture with Müller cells (Fig. 3). However, if we analyse the increase of survival in co-cultures versus homotypic ones we found a increase of + 42.1 ± 7.1% in normoxic condition and + 55.8 ± 11.4% in hypoxic condition when Müller glial cells are present. These data indicate that hypoxia compromises the viability of RGC and that Müller cells release (a) neuroprotective factor(s), which is (are) able to compensate apoptotic loss of RGC.

### PEDF Accounts for the Neuroprotective Müller-Cell Derived Activity

**Fig. 4:**
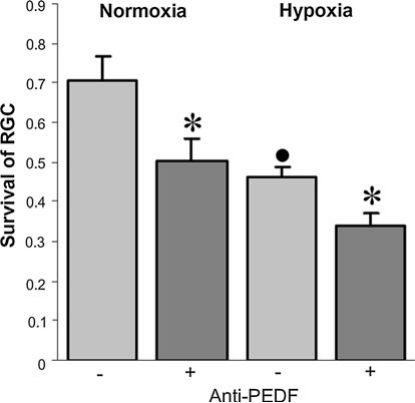
Effect of neutralizing anti-PEDF antibody Müller cell-promoted RGC survival in co-cultures is suppressed by a neutralizing anti-PEDF antibody in both normoxic and hypoxic conditions (*n* = 4; anti-PEDF vs. control cultures, *P < 0.05; control hypoxic versus normoxic control cultures, ^•^ *P* < 0.05). Co-cultures were maintained in the presence of an anti-PEDF antibody (+) or normal goat immunoglobulin in control cultures (−)

To determine whether Müller-cell derived PEDF contributes to the observed neuroprotection, a neutralizing anti-PEDF antibody was added to the cultures (Fig**.** 4). We previously used this antibody to neutralize PEDF-mediated functions in retinal endothelial cells [9] and employed it here at 3 μg/ml to fully deplete the glia-conditioned medium from PEDF. Under normoxic and hypoxic conditions, the increasing survival rate induced by Müller-cell derived soluble mediators (71 ± 6% and 46 ± 2% respectively) was significantly impaired by the antibody treatment of co-cultures with a resulting survival rate of 50 ± 6% and 34 ± 3% in normoxia and hypoxia respectively. In homotypic RGC cultures, there was no effect of antibody treatment on RGC survival, which amounted to a level similar to that in the absence of PEDF, indicating the absence of antibody toxicity toward RGC (data not shown). Overall, these results suggest that PEDF represents an essential soluble mediator contributing to the survival-enhancing activity of Müller cell-derived factors.

## Discussion

RGC are subjected to neuronal death under various pathogenic conditions. Among those is glaucoma, a chronic neurodegenerative disease in which RGCs ultimately die from apoptosis in response to complex and multifaceted insults including ischemia, which can be due to elevated intraocular pressure, hypoxia, glutamate excitotoxicity, and oxidative stress [40]. PEDF has been show to exhibit neuroprotective properties on RGC in case of glutamate excitotoxicity [17] and of elevated intraocular pressure [22, 30, 31], indicating its potential use in the development of therapeutic strategies to fight glaucoma, the second leading cause of blindness after cataract worldwide.

Here we demonstrate for the first time the direct neuroprotective role of PEDF on RGC. We compared mRNA levels of this factor expressed by RPE and glia cells and showed that PEDF is a glia-secreted factor that protects RGC from a hypoxic insult. Altogether, our results suggest that PEDF could constitute an essential survival factor for RGC when released by Müller glial cells in response to a pathological state. Neuroprotective functions of PEDF have largely been described in various neuronal types. PEDF prevents glutamate-induced apoptotic cell death in the CNS in general [15, 16, 41] and in the retina in particular [42]. In this study, we are underlining the contribution of PEDF originating from Müller glial cells in the neuroprotection of RGC. Apart form the RPE, the canonical source of PEDF in the eye, immunohistological analysis of mouse retina clearly showed that PEDF signal was maximal in Müller glia endfeet. We cannot fully rule out the fact that some of the PEDF we detected in glia cell may have come from a RPE secretion taken up by Müller glial cells rather than from a synthesis in situ, however this is highly unlikely considering the similar expression level of PEDF mRNA in those two cell types in vitro. Even if we assessed PEDF transcript levels in cultured cells rather than cells originating from in vivo material it is reasonable to assume that both retinal glial (Müller) and RPE cells produce a comparable amount of PEDF in situ. PEDF synthesized by Müller glial cells may be secreted in vivo at glial cell endfeet*,* in the vicinity of the RGC bodies, and may counterbalance a neurodegenerative insult targeting this cell type. As recently shown [33], when injected in the subconjunctiva, fluorescent serpin is mainly found in photoreceptor layer and do not reach the RGC layer after 24 h. This suggests that PEDF secreted at the RPE layer could barely have a neuroprotective effect on RGC and our present results using co-culture and PEDF neutralizing antibody strongly support the hypothesis of a Müller glial contribution in this mechanism.

Interestingly co-culture with Müller glial cell under hypoxic condition did not fully protect RGC. The survival was 42.2 ± 3.5% in hypoxic co-culture versus homotypic RGC culture while co-culture improved the survival at 65.9 ± 4.4% in normoxic condition. The apparent reduced efficiency of Müller glial neuroprotection may be linked to changes in the level of released survival factors and/or to the noxious effect of additional factors secreted by reactive glial cell; forming the Janus face of the reactive glia [43, 44]. However if one focus on the cell survival improvement, the co-culture with Müller glial cells increased RGC survival in the same order of magnitude in normoxic and hypoxic conditions. Efficiency on neuroprotection in the co-culture was also found similar to the growth factor addition in the medium using PEDF at 25 ng/ml. This optimal neuroprotection under hypoxia obtained with a PEDF concentration of 25 ng/ml is very similar to the optimal PEDF concentration to achieve neuroprotection of RGC under glutamate insult, with an EC50 value at 13.5 ng/ml [17]. Last but not least, neutralizing anti-PEDF added to co-cuture reduced the RGC survival to its minimal values. Altogether our results suggest that PEDF is therefore one of the released factors secreted by glia cell that could directly protect RGC in case of various insults. In addition, we have previously shown that mRNA expression of PEDF is modulated in Müller glial cells by hypoxic condition [8, 10]. However, at the protein level, PEDF secretion was found similar under normoxic condition and strong hypoxia [10]. Therefore, the precise regulation of this factor by glial cell in vivo following a hypoxic stress has still to be analyzed as several studies underlined by their apparent discrepancy the complex regulation of PEDF expression in this pathological condition [8, 10, 45–47].

As PEDF released from glial cell has been shown here to be as efficient as CNTF treatment, understanding its regulation and being able to stimulate its secretion by the Müller glial cell in vivo would consist in a promising strategy to achieve neuroprotection of the RGC by their close glial partner. Müller glial cells are indeed the principal glial type of the retina and span the entire thickness of this tissue, ensheathing all neurons. This glial cell type can be called a “retinal swiss knife” that achieves a multitude of functional interactions with neurons under normal and pathological conditions [43]. In response to virtually all type of insult (retinal detachment, glutamate toxicity, hypoxia) Müller glial cells undergo an early step of reactive gliosis [44] characterized by changes in their intermediate filament composition [48], activation of extracellular signal-regulated kinase (ERKs) [49], and release of neuroprotective factors [10, 50, 51]. During this early step, gliosis is thought to be mainly neuroprotective and may reflect an attempt to rescue retinal neurons from further damage. By contrast, later gliotic events such as dedifferentiation, glial proliferation, and glial scar formation, largely contribute to neuronal cell death. The balance between beneficial and detrimental events in reactive gliosis is therefore a critical point in retinal neuroprotection, and influencing this balance “in situ” could lead to therapeutic strategies in fighting various retinopathies. Interestingly, high levels of survival factor(s), normally secreted by reactive Müller glial cells, are able to keep the gliosis under control by suppressing the detrimental proliferative response of Müller glial cells [52]. PEDF itself has been shown to preserve the expression of glutamine synthetase level, to downregulate GFAP expression in reactive gliosis, and to prevent the degenerative glial response in retina by reducing the inflammatory state of Müller glial cells [31, 53, 54]. Increasing the PEDF content specifically in Müller glial cells through transfection [55] or transplantation [56] may result in original strategies to take advantage from its neuroprotective properties towards RGC, along with the global beneficial response of glia, while avoiding the detrimental consequence of a massive gliosis in response to neurodegenerative stimuli.
